# Emergency pulpotomy in relieving acute dental pain among Tanzanian patients

**DOI:** 10.1186/1472-6831-6-1

**Published:** 2006-01-21

**Authors:** Joachim W Nyerere, Mecky I Matee, Elison NM Simon

**Affiliations:** 1 Department of Restorative Dentistry, School of Dentistry, Muhimbili University College of Health Sciences, P. O. Box 65014, Dar Es Salaam, Tanzania; 2 Department of Microbiology and Immunology, School of Medicine, Muhimbili University College of Health Sciences, P. O. Box 65014, Dar Es Salaam, Tanzania; 3 Department of Oral Surgery and Oral Pathology School of Dentistry, Muhimbili University College of Health Sciences, P. O. Box 65014, Dar Es Salaam, Tanzania

## Abstract

**Background:**

In Tanzania, oral health services are mostly in the form of dental extractions aimed at alleviating acute dental pain. Conservative methods of alleviating acute dental pain are virtually non-existent. Therefore, it was the aim of this study to determine treatment success of emergency pulpotomy in relieving acute dental pain.

**Methods:**

*Setting: *School of Dentistry, Muhimbili National Hospital, Dar es Salaam, Tanzania.

*Study design: *Longitudinal study.

*Participants: *180 patients who presented with dental pain due to acute irreversible pulpitis during the study period between July and August 2001.

*Treatment and evaluation: *Patients were treated by emergency pulpotomy on permanent posterior teeth and were evaluated for pain after one, three and six week's post-treatment. Pain, if present, was categorised as either mild or acute.

**Results:**

Of the patients with treated premolars, 25 (13.9%) patients did not experience pain at all while 19 (10.6%) experienced mild pain. None of the patients with treated premolars experienced acute pain. Among 136 patients with treated molars 56 (31%) did not experience any pain, 76 (42.2%) experienced mild pain and the other 4 (2.2%) suffered acute pain.

**Conclusion:**

The short term treatment success of emergency pulpotomy was high being 100% for premolars and 97.1% for molars, suggesting that it can be recommended as a measure to alleviate acute dental pain while other conservative treatment options are being considered.

## Background

In Tanzania, it is estimated that over 85% of dental patients seek dental care due to painful tooth conditions, and that over 90% of the treatment rendered is tooth extraction [1]. A total of 159,660 extractions were conducted in 1993 and 99,636 extractions in 1995 in the 20 regions of Tanzania, the majority of them due to dental caries [2]. These extractions are unjustifiably too many and therefore the situation needs to be rectified. Thus, any strategy aiming at improving emergency oral health services in Tanzania should at least strive at minimising the number of undue extractions performed.

Previous studies [3-6] have shown that emergency pulpotomy is very effective in relieving acute dental pain caused by acute pulpitis. The emergency pulpotomy procedure includes the removal of the coronal pulp exposed by caries, cleaning the cavity, dressing the access cavity and filling with a temporary restoration. This can be easily done in most regional and district government dental clinics as well as in private clinics in Tanzania, potentially resulting in reduction of the number of extractions. However, the level of success of emergency pulpotomy undertaken in a setting like ours, with minimal dental resources, has yet to be determined, making it difficult to estimate the potential benefits.

Therefore, the aim of this study was to determine the level of success of emergency pulpotomy in relieving acute dental pain resulting from pulp exposure due to dental caries.

## Methods

A total of 180 patients aged ≥ 15 years, who had dental pain due to acute pulpitis, which under current circumstances would have been extracted, were included after obtaining an informed consent. Enrolment was done consecutively, including all eligible patients who were attended at the Restorative Department of the Muhimbili National Hospital (MNH) during the study period between July and August 2001. Patients were excluded if found to have any of the following: Teeth that had grossly destroyed crowns and/or clinical or radiographic signs of pulp degeneration or third degree mobility.

During the first visit to the clinic emergency pulpotomy was done on premolars or molars pending an alternative conservative treatment such as root canal treatment.

Pulpotomy involved administration of local anaesthesia (Lignocaine 2 cc with epinephrine or, for hypertensive patients, without epinephrine) followed by the removal of the coronal portion of the pulp using a round carbide bur. Haemostasis was achieved by pressing a sterile cotton pellet against the site of amputation for 2–3 minutes. The pulp chamber was irrigated with 2.5% sodium hypochlorite and was dried with a sterile dry cotton pellet. A sterile cotton pellet moistened with eugenol solution was then placed against the remaining pulp tissue. The access cavity was sealed with reinforced zinc oxide eugenol cement and the occlusion checked. All the clinical work was done by JWN.

Evaluation of treatment included clinical and radiographic examination done after one, three and six week's post-treatment using evaluation forms. The final outcome was taken after results of evaluation on third visit. Presence of acute pain, apical periodontitis (acute or chronic), tooth mobility, radiographic evidence of pulp degenerative changes or loss of vitality was regarded as an indicator of a failed treatment. Patients were also questioned on the presence or absence of pain and the degree of pain experienced. Pain was regarded as being mild if it was not discomforting or prevent the patient from sleeping and did not require the prescription of analgesics. Acute dental pain was that pain that was difficult to bear and was very disturbing to the patient necessitating the use of analgesics or further intervention. All successful cases were scheduled for root canal treatment and restoration at appropriate intervals.

### Data analysis

Data was entered into computer and analysis was done using a calculator by calculating percentages of the different outcomes.

### Ethical issues

Verbal consent was obtained from all the participants and ethical clearance was obtained from the Muhimbili University College of Health Sciences (MUCHS) ethical committee.

## Results

There were 180 patients who had teeth that were treated with emergency pulpotomy, of whom majority (69.4%) were less than 40 years of age (Table 1). In each patient only one tooth was treated.

**Table 1:** Distribution of patients treated by emergency pulpotomy according to age and tooth type.

Age (yrs)	Mandibular				Maxillary				Total	
	Premolars		Molars		Premolars		Molars			

	n	%	n	%	n	%	n	%	n	%
15 – 20	6	3.3	20	11.1	3	1.6	12	6.7	41	22.8
21 – 30	2	1.1	17	9.4	7	3.9	15	8.3	41	22.8
31 – 40	2	1.1	17	9.4	8	4.4	16	8.9	43	23.9
41 – 50	3	1.6	14	7.8	5	2.8	8	4.4	30	16.6
51 – 60	3	1.6	7	3.9	2	1.1	3	1.6	15	8.3
> 60	1	0.6	5	2.8	2	1.1	2	1.1	10	5.6

Total	17	9.4	80	44.4	27	15	56	31.2	180	100

There were a total of 44 premolars that were treated out of which 27 were maxillary and 17 mandibular. Out of 136 treated molars, 56 were maxillary and 80 mandibular (Table 1). After emergency pulpotomy 18 maxillary premolars which formed 10% of all the treated teeth and 7 (3.9%) mandibular premolars did not cause any pain at all. However, 9 (5%) maxillary and 10 (5.6%) mandibular premolars caused mild pain (Table 2). None of the premolars caused acute pain. There was no pain in 22 (12.2%) maxillary and 34 (17.8%) mandibular molars while 32 (17.8%) maxillary and 44 (24.4%) mandibular molars caused mild pain. Of the molars that caused acute pain two (1.1%) were maxillary and 2 (1.1%) mandibular.

**Table 2:** Distribution of patients treated by emergency pulpotomy according to level of pain experienced.

Tooth type	Mandibular				Maxillary				Total	
	Premolars		Molars		Premolars		Molars			

	n	%	n	%	n	%	n	%	n	%
No pain	7	3.9	34	17.8	18	10	22	12.2	81	45
Mild Pain	10	5.6	44	24.4	9	5	32	17.8	95	52.8
Acute pain	0	0	2	1.1	0	0	2	1.1	4	2.2

Total	17	9.4	80	44.4	27	15	56	31.2	180	100

## Discussion

This study was prompted by the unjustifiably large number of extractions in Tanzania, which forms over 90% of all the dental treatments offered [2]. The reasons that have been advanced for this situation include; emergency relief of severe acute dental pain, low cost (0.5 and US$1.00), lesser number of visits to the clinic, short time to carry out the procedure, and modest requirements in terms of personnel and equipment. In our institution eugenol application is the standard practice in endodontics. Our study was done in this context. The use of eugenol and/or zinc oxide-eugenol in pulpotomy has also been practiced by others [7].

In this study, we observed a hundred percent success rate in premolars and ninety eight percent in molars, which compares with success rates of between (53% and 99%) reported in other studies [8,9]. This level of success is remarkable given the high HIV seroprevalence (9.4%) of this patient population, the rather low level of oral hygiene and lack of awareness [10]. Such factors could predispose to high rates of infection and therefore diminish the chances of success. Differences in success between this and the other studies [9] could be attributed to undiagnosed, subclinical inflamed pulp, while long-term failure may be associated with micro-leakage of the temporary restorative material.

## Conclusion

With this success rate it can be suggested that emergency pulpotomy if widely employed could potentially reduce the present number of extracted teeth in Tanzania and probably other neighbouring countries with similar situations by approximately 90%. In Tanzania dental personnel at the district and regional levels could do pulpotomy as an emergency measure. However, this will require the dental therapists, who carry out the bulky of primary oral health services to be re-trained [11]. These efforts need to be matched with education to the Tanzanian public on the expanded range of treatment possibilities in order for them to make informed treatment choices.

## Competing interests

The author(s) declare that they have no competing interests.

## Authors' contributions

JWN took part in data collection, data handling and preparation of the manuscript, MIM participated in organising and preparation of the manuscript, ENMS took part in data entry and the preparation of the manuscript.

## Pre-publication history

The pre-publication history for this paper can be accessed here:


